# A brief overview of field testing and commercial application of transgenic trees in China

**DOI:** 10.1186/1753-6561-5-S7-O63

**Published:** 2011-09-13

**Authors:** Meng-Zhu Lu, Jian-Jun Hu

**Affiliations:** 1 State Key Laboratory of Forest Genetics and Tree Breeding, Chinese Academy of Forestry, Beijing 100091, China

## 

Since the first report on transformation of *Bt* gene into *Populus nigra* was published in 1989, transgenic approaches have been widely used in breeding trees for high tolerance to insects and environmental stresses in China. Notably, field testing of the transgenic poplar with *Bt* was performed in 1994 and permitted to be commercialized as the first commercialized transgenic tree in the world. Up to now, nearly 50 species have been used as recipients for genetic transformation including poplar, birch, locust tree, walnut, aimed to improve their tolerance to insects and diseases, environmental stresses, prolonged storage lifetime, wood property, flowering time control, etc..

On May 5, 2006, the State Forest Administration (SFA) launched its first regulatory framework on “Management measures on the inspection and permit of forest genetic engineering” taken effect on July 1, 2006. It states that small-scale field testing, environmental release and pilot production test of each transgenic line are required before its permit of commercialization, with each phase must be evaluated by the experts. “A technical guideline on safety evaluation of the transgenic forest plants and their products” was published by SFA on June 4, 2007, which provides a standard procedure for molecular breeders to follow in order to commercialize their transgenic trees.

To the year 2010, 128 field trials have been granted by SFA, 84 of which belong to tree species while 44 to grass species. Thirty three of them were granted to transgenic poplars, with tolerance to insects and diseases, drought and salt stresses, altered wood property, etc. , and 25 to locust tree. Transgenic *Populus tomentaosa* with antisense *CCoAOMT* (coding for a key enzyme involved in lignin monomer biosynthesis) , transgenic *Robinia pseudoacacia* with *BADH*, *Sophora japonica* with *RD29A* and *P. nigra* with *Bt* are under environmental release testing.

The transgenic poplar plantation has increased to 450 hm^2^ this year since two *Bt* transgenic poplars were commercialized in 2001 in China. The transgenic poplar plantations have effectively inhibited the fast-spread of target insects and significantly reduced the times of insecticide application on poplar plantation. The transgenic *Populus nigra* was also used in hybridization with non-transgenic *P. deltoides* as an insect-resistant source for breeding new hybrid clones.

